# DOD-Boost: a temporal and distribution-optimized deep boosting framework for solar radiation modeling

**DOI:** 10.1038/s41598-025-19157-6

**Published:** 2025-09-29

**Authors:** İlker Mert

**Affiliations:** Osmaniye Vocational School, Electric & Energy Department, Osmaniye Korkut Ata University, Osmaniye, 80010 Turkey

**Keywords:** Hybrid deep learning, Weibull distribution, Solar radiation forecasting, Optimization algorithms, Temporal modeling, Engineering, Mathematics and computing

## Abstract

This study proposes hybrid solar radiation temporal modeling approaches to support the design of clean energy systems using deep learning techniques and statistical distribution fitting. Solar radiation data are analyzed using a probability distribution to determine whether they follow a known statistical pattern, focusing on total solar radiation on a tilted surface (MJ/m^2^) ($\:{H}_{T}$). Maximum likelihood estimation (MLE), whale optimization algorithm (WOA), and particle swarm optimization (PSO) are used to optimize the process of estimating probability distribution parameters. Subsequently, the cumulative distribution function (CDF) is constructed, and a particular distribution profile is applied to replace the inherent randomness in $\:{H}_{T}$ data during the preparation phase of estimation model inputs. In the next step, innovative hybrid $\:{H}_{T}$ temporal modeling approaches based on CDF are developed using long short-term memory networks (LSTMs), gated recurrent units (GRUs), and extreme gradient boosting (XGBoost) algorithms. Model results are evaluated through Jensen-Shannon divergence (JSD) analysis. Thus, the DOD-Boost framework is established. According to the findings from comprehensive analyses, DOD-Boost models that integrated a modeling approach for $\:{H}_{T}$, optimization techniques, data preprocessing strategies, and temporal modeling achieved highly accurate predictions. Among all tested models, the Weibull (WOA) – LSTM – XGBoost model achieved the best distributional accuracy, with the lowest JSD value of 0.0084. The JSD metric was prioritized as it provides a more comprehensive assessment of performance by measuring the similarity of the predicted and actual data distributions, which is more informative than simple point predictions for energy planning. Consequently, this study provides a transferable hybrid model for PV-based energy planning that can also be used in developing countries.

## Introduction

Solar radiation, $H$ (MJ/m^2^), is a critical part of clean energy production, and its variability depends on atmospheric, geographical, and temporal factors. It is important to predict this variability and optimize solar energy systems accordingly. In this way, efficient system use, and stable energy supply become more realistic and achievable expectations. Short-term and long-term $H$ (MJ/m^2^) forecasts for energy companies and power system control units need greater accuracy. Recently, in the context of the variable nature of $H$ (MJ/m^2^) data, various machine learning (ML) and deep learning (DL) techniques have been investigated for $H$ (MJ/m^2^) prediction. These techniques can be classified into five groups: Artificial Neural Networks (ANN, MLP, FFBP), Support Vector Regression (SVR), ANFIS and ANFIS Models with Optimization, DL Models (LSTM, BiLSTM, GRU, RNN, CNN) and Tree-based and instance-based models (Decision Tree (DT), Random Forest (RF), and KNN)^1–6^. Lara-Benítez et al. recommended MLP and CNN, while Alrashidi et al. highlighted the performance of SVR-GOA^2,6^. Huang et al. emphasized the optimized ANFIS-GOA, whereas LSTM-based methods, RNN, and CNN show remarkable performance in many regions^7,8^. Tree-based and instance-based models were used for comparison in some studies but generally did not give the best results. In summary, while optimization-enhanced ANFIS and DL hybrids (BiLSTM-GRU, LSTM-GP) are generally the most successful methods, classical methods such as SVR and ANN are still in use. However, their performance is limited when not integrated with optimization algorithms (e.g., GOA, PSO, SSA). Methods with spatial modeling capabilities, such as CNN and transformer-based models, can deliver remarkable improvements—especially when combined with other models (MLP-CNN, LSTM-CNN)^6^. The intrinsic stochastic character of solar radiation, as formed by meteorological, geographical, and temporal circumstances, presents considerable challenges for reliable forecasting. Michael et al. (2024) analyzes BiLSTM and a hybrid BiLSTM-GRU DL model to address these challenges^9^. The proposed models employ Bayesian optimization for hyperparameter tuning. The analysis is based on SoDa dataset within the scope of power system optimization with energy storage systems. The results of these analyses demonstrate that the BiLSTM-GRU model, enhanced with dropout, significantly improved forecasting accuracy and reduced errors (RMSE = 1.41, MAE = 0.91). Cornejo-Bueno et al. (2019) evaluate the performance of several regression techniques (Support Vector Regression (SVR), Multi-layer Perceptron (MLP), Extreme Learning Machine (ELM), and Gaussian Process (GPR)) in a problem of global solar radiation (GSR) estimation from satellite data. Although the cloud index, a clear-sky solar radiation model, and several reflectivity values are considered input variables, hourly GSR is used as the target variable in all models. Results show that ML regressors obtained reliable GSR estimation using satellite measurements^1^. Real-time solar energy forecasting and short-term are all encompassed in a unified tool that combines the Pearson correlation coefficient and the deep ANN implementation by Jebli et al. (2021). Because of its performing deep computation using several learning layers, ANN was considered superior to all of other models for daily solar energy prediction^10^. Results from this investigation indicate that accurately appropriating the stochastic dynamics of solar radiation is perhaps a challenging open field research question requiring advancements in modeling methodologies in the future. Yadav et al. (2025) compared RBFNN, LSTM, MNN, and Transformer models for daily global solar radiation prediction using meteorological stochastic variables, showing that the Transformer model achieved the highest accuracy with a 1.98% error rate^11^. Rajagukguk and Lee (2025) employed CatBoost-based explainable machine learning techniques to predict direct (DNI) and diffuse (DHI) components from GHI data, interpreting input feature importance through SHAP analysis^12^. Attya et al. (2025) integrated tabular data with satellite imagery in a hybrid framework, combining noise reduction, pixel inpainting, and LSTM-based methods to significantly improve solar radiation prediction accuracy^13^.

Considering the aforementioned studies, it is possible to say that $\:H$ (MJ/m²) estimation requires the use of more flexible and powerful approaches beyond classical estimation methods. In this sense, a hybrid approach that combines the strengths of statistical modeling and DL, called the distribution-optimized deep boosting framework (DOD-Boost), can be a more effective solution. The present study aims to develop hybrid models for temporal modeling $\:H$ (MJ/m^2^) by combining DL techniques with statistical distribution fitting^14^. For this purpose, as a preliminary step, the Weibull distribution (WD) is utilized to analyze the statistical properties of $\:H$ (MJ/m^2^) data. The distribution is optimized to better understand the natural structure of the $\:H$ (MJ/m^2^) data and to enrich the inputs of the temporal part of the hybrid models. Optimization will be done using Deterministic Maximum Likelihood Estimation (MLE) and meta-heuristic Whale Optimization Algorithm (WOA) and Particle Swarm Optimization (PSO). Thus, the model can better capture the basic properties of $\:H$ (MJ/m^2^). The underlying structural patterns of the data have become possible to learn more accurately with the Weibull cumulative distribution function (WeibullCDF), which is improved with optimized parameters. Then, hybrid temporal modeling architectures are developed that integrate WeibullCDF with Long Short-Term Memory (LSTM), Gated Recurrent Unit (GRU) and Extreme Gradient Boosting (XGBoost) algorithms. Thus, the DOD framework is created. LSTM is an effective temporal DL model for long-term dependencies in time series data ($\:TSD$)^15^. GRU can be used to capture long-term dependencies in $\:TSD$, like LSTM^16^. The GRU is particularly suitable for the estimation of data that varies over time, such as $\:H$ (MJ/m^2^), due to its ability to learn complex patterns in sequential data. However, GRU uses a minimal number of training parameters. Therefore, it uses less memory and performs faster computing than LSTM. XGBoost is an optimized version of the gradient boosting algorithm^17^. The XGBoost model, which performs well in both regression and classification problems, is preferred to correct errors in LSTM and GRU’s predictions and achieve more accurate results. While studies in^1–13^ have focused on either point forecasts ($\:{R}^{2}$, RMSE, MAE, etc.) or distributional modeling of solar radiation, no one has successfully integrated both approaches.

Despite extensive research, a critical gap remains in the literature: current solar forecasting models typically excel in either point accuracy or distribution fidelity, but not both. This study bridges this gap by proposing the novel Distribution-Optimized Deep Boosting (DOD-Boost) framework. Its primary contribution is a unified methodology that integrates statistical distribution optimization (via Weibull CDF with MLE/PSO/WOA), deep temporal learning (LSTM/GRU), and ensemble boosting (XGBoost) to simultaneously achieve superior point forecasts and unparalleled distributional accuracy, as measured by Jensen-Shannon Divergence (JSD). Furthermore, this work reveals and analyzes the inherent trade-off between these objectives, providing a clear guide for model selection based on application needs. The proposed framework offers a significant advance for robust, probabilistic solar forecasting, which is particularly vital for resource-limited settings.

## Materials and methods

DOD-Boost framework offers accurate $\:H$ (MJ/m^2^) estimates and guided decision-making on photovoltaic system performance, which is especially important for sustainable energy planning in countries with limited resources. The methodology consists of four main stages: data collection, model development, evaluation, and estimation. $\:H$ (MJ/m^2^) data are obtained from the Turkish State Meteorological Service (TSMS). For this study, data from the Karaman province $\:\left({37}^{^\circ\:}{12}^{{\prime\:}}N,\:{33}^{^\circ\:}{17}^{{\prime\:}}E\right)$ covering the period from 01.01.2009 to 31.12.2009 are used, specifically because this time frame does not contain any missing data. The dataset is subsequently split into training and test datasets with a chronological split ratio of 80–20%, respectively. The daily $\:H$ (MJ/m^2^) data are modeled using the Weibull distribution. The distribution parameters (s, m) are iteratively estimated using MLE, WOA, and PSO, resulting in three distinct parameter pairs. Using these pairs, WeibullCDF values are calculated. These values, along with the observed $\:H$ (MJ/m^2^) data, are input to the LSTM network. Accordingly, as part of the DOD framework, three hybrid Weibull-LSTM models are developed based on the parameter sets obtained from MLE, WOA, and PSO. In these models, the observed $\:H$ (MJ/m^2^) value is used as the target variable. The same methodology is applied using the GRU network, resulting in three additional Weibull-GRU hybrid models. $\:H$ (MJ/m^2^ is estimated both in terms of temporal dynamics as well as statistical distribution characteristics using these hybrid models. Including WeibullCDF values improves the LSTM and GRU models’ ability to learn, contributing to a more accurate representation of $\:H$ (MJ/m^2^). The steps necessary for the development of the DOD-Boost framework are outlined below. For the purpose of increasing model robustness, an ensemble learning decision tree algorithm, XGBoost, was adopted. XGBoost has been integrated into six models due to its robustness in solving regression and classification problems. The outputs of the Weibull - LSTM models— namely, the LSTM predictions and the corresponding WeibullCDF values—are fed into XGBoost to create more powerful hybrid configurations. In this stage, the XGBoost model is trained to predict the actual $\:H$ (MJ/m^2^) values. Through its feature importance mechanism, XGBoost identifies which input—LSTM prediction or WeibullCDF—contributes more significantly to the final output, allowing it to effectively utilize both statistical and temporal features. Consequently, incorporating XGBoost contributes to the model’s interpretability and prediction performance. Three additional Weibull-LSTM-XGBoost hybrid models are developed using the MLE, WOA, and PSO-based parameters, respectively. Similarly, three Weibull-GRU-XGBoost hybrid models are created by applying the same approach with the GRU network. Estimating the $\:H$ (MJ/m^2^) is conducted using 12 hybrid models, including LSTM, GRU, and XGBoost architectures, and WeibullCDF based on WD optimized by MLE, WOA, and PSO. Using this comprehensive framework, temporal modeling and non-linear relationship modeling are supported, as well as feature selection through importance analysis, regularization, and hybrid modeling, thus improving $\:H$ (MJ/m^2^) prediction. The next step of this framework involves comprehensive performance evaluation. Kernel Density Estimation (KDE) is used to construct the probability density functions of the predicted and actual $\:H$ (MJ/m^2^) data, enabling distribution-level comparison. The Jensen-Shannon Divergence (JSD)—a symmetric and stable distance metric—is then used to assess the dissimilarity between the predicted and observed distributions^18^. This analysis is instrumental in evaluating the degree to which the model’s predicted values correspond to the actual data. The outputs of the best models are analyzed to determine whether they fit with the observed data. A low JSD value shows that the distributions are comparable, while a high value suggests notable differences.

**Fig. 1:**
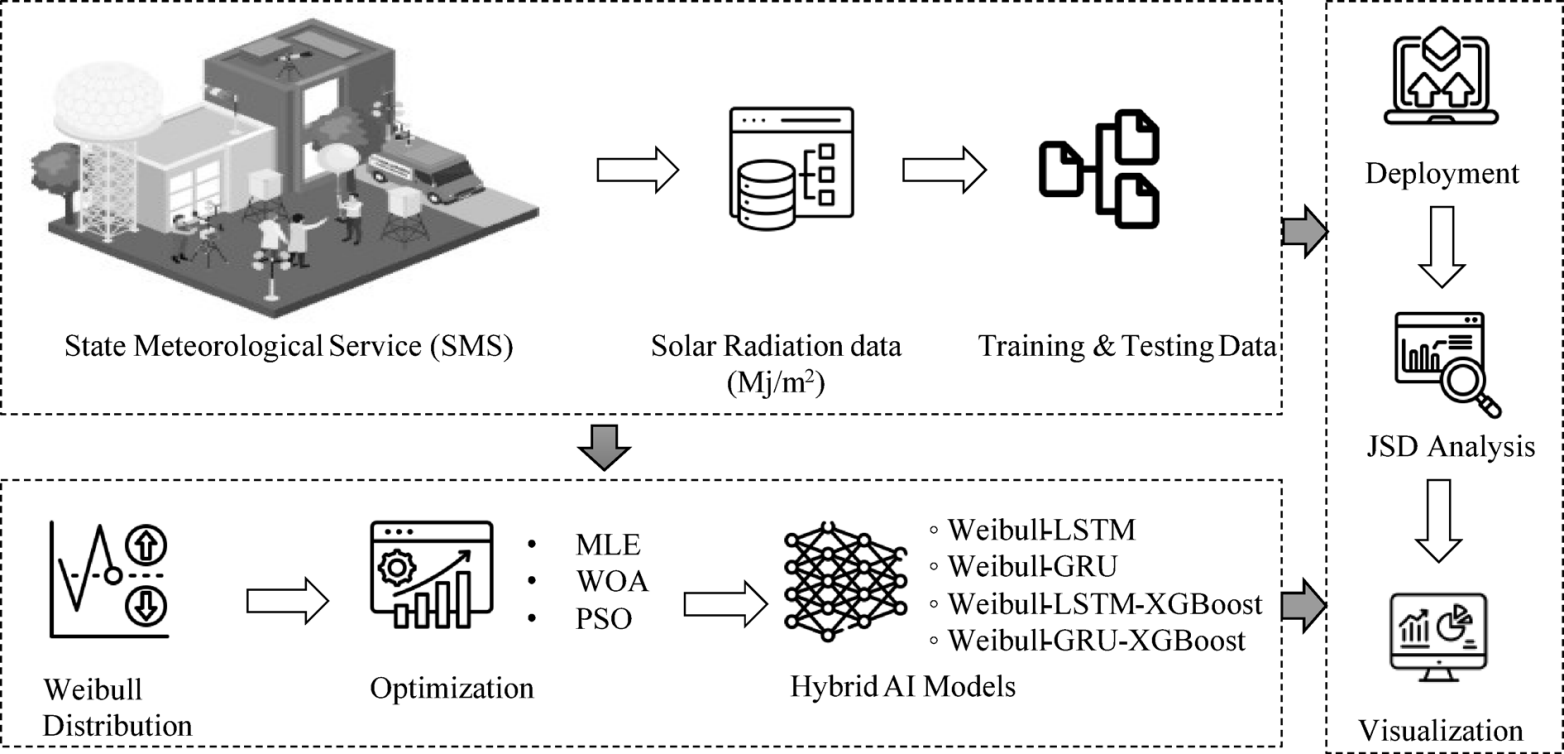
Representative DOD-Boost block diagram.

The processes conducted during this study are depicted representatively in Fig. 1.

### Weibull distribution (WD)

The WD has two parameters. In the univariate two-parameter Weibull distribution, *m* (shape parameter) indicates the shape of the distribution. $\:s$ (scale parameter) defines the scale of the distribution. The corresponding cumulative distribution function (CDF) and probability density functions (pdf) are^19^:

1 $$\:g\left(x\right)=1-\text{e}\text{x}\text{p}\left(-{\left(\:\frac{x}{s}\:\right)}^{m}\right)$$

2 $$\:G\left(x\right)=\frac{m}{s}{\left(\:\frac{x}{s}\:\right)}^{m-1}\:\text{e}\text{x}\text{p}\left(-{\left(\:\frac{x}{s}\:\right)}^{m}\right)$$

The expected daily $\:H$ (MJ/m^2^) value can be calculated using the WD^20^:

3 $$\:{H}_{Weibull}=s\:{\left(-\text{l}\text{n}(1-p)\right)}^{\frac{1}{m}}$$

where $\:p$ is the probability value (here $\:p=0.5)$.

For an inclined PV panel, $\:{H}_{T}$ is the total radiation (MJ/m^2^) and consists of direct, isotropic diffuse and reflected components^21^.

4 $$\:{H}_{T}=H\left(1-\frac{{H}_{d}}{H}\right){\stackrel{-}{R}}_{b}+{H}_{d}\left(\frac{1+cos\beta\:}{2}\right)+{H}_{\rho\:}\left(\frac{1-cos\beta\:}{2}\right)$$

where $\:H$ is defined as total solar radiation (MJ/m^2^), $\:{\stackrel{-}{R}}_{b}$ is defined as the ratio of the daily direct radiation falling on the inclined surface to the daily direct radiation falling on the horizontal surface. $\:{H}_{d}$ is diffuse component of total radiation. $\:{H}_{\rho\:}$ is reflected components of total radiation.

5 $$\:{\stackrel{-}{R}}_{b}=\frac{\text{cos}\left(\varphi\:-\beta\:\right)cos\delta\:sin{\omega\:{\prime\:}}_{s}+\left(\frac{\pi\:}{180}\right){\omega\:{\prime\:}}_{s}sin\left(\varphi\:-\beta\:\right)sin\delta\:}{\text{cos}\varphi\:cos\delta\:sin{\omega\:}_{s}+\left(\frac{\pi\:}{180}\right){\omega\:}_{s}sin\varphi\:sin\delta\:}$$

where $\:\beta\:$, $\:\varphi\:$, and $\:\delta\:$ represent the slope angle (degrees), latitude angle (degrees), and declination angle (degrees) of the surface. $\:{\omega\:{\prime\:}}_{s}\:$is the sunset hour angle on the tilted surface (degrees). $\:{\omega\:}_{s}$ is sunset hour angle on a horizontal surface (degrees).

### Maximum likelihood estimation (MLE)

Estimating the parameters of the WD using the Maximum Likelihood Estimation (MLE) can be a statistically powerful approach. MLE is a method based on estimating the parameters in a way that maximizes the likelihood of the observed data. The likelihood function can be given using the Weibull PDF as follows^22^:

6 $$\:L\left({D}_{1},\dots\:,{D}_{n};s,m\right)\prod\:_{i=1}^{n}\frac{m}{s}{\left(\frac{D}{s}\right)}^{m-1}exp\left(-{\left(\frac{D}{s}\right)}^{m}\right)$$

where $\:D$ represents data points. Estimates for $\:s$ and $\:m$ parameters can be given as follows by taking the derivative of the log-likelihood function.

7 $$\:s={\left[{n}^{-1}\sum\:_{i=1}^{n}{D}_{i}^{c}\right]}^{1/m}$$

8 $$\:m=\left(\frac{\sum\:{D}_{i}^{c}ln{D}_{i}}{\sum\:{D}_{i}^{c}}-\frac{1}{n}\sum\:_{i=0}^{n}ln{D}_{i}\right)$$

### Particle swarm optimization (PSO)

PSO is an evolutionary optimization algorithm inspired by the behavior of bird flocks and fish schools in nature. PSO can use to estimate the $\:s$ and $\:m$ parameters of the Weibull distribution. When estimating Weibull parameters, negative log-likelihood (NLL) can be used as the objective function in the estimation process with the maximum likelihood method (MLE)^23^.

9 $$\:NLL\left(s,m\right)=-\text{l}\text{o}\text{g}{P}_{T}\left(y|s,m\right)=nlog\left(\frac{{s}^{m}}{m}\right)-\left(m-1\right)\left(\sum\:_{i=1}^{n}log{y}_{i}\right)+\sum\:_{i=1}^{n}{\left(\frac{{y}_{i}}{s}\right)}^{m}$$

Then PSO tries to minimize NLL by optimizing $\:s$ and $\:m$ parameters.

10 $$\:\underset{s,m}{\text{min}}NLL\left(s,m\right)$$

PSO tries to find the optimum parameters using the set of particles. For this purpose, firstly random bb and cc values are set, and then the speed ($\:v$) and position ($\:x$) are determined for each particle. After calculating the NLL for each particle, the particles with the lowest NLL value are considered as the best solution candidates. Let the $\:x$ and $\:v$ of particle $\:k$ be denoted by $\:{x}_{k}=\left({x}_{k,1},\dots\:,{x}_{k,d}\right)$ and $\:{v}_{k}=\left({v}_{k,1},\dots\:,{v}_{k,d}\right)$, respectively ($\:k=\text{1,2},\:\dots\:,\:{p}_{n})$^24^.

Each particle moves in the direction of both its best position and the best position of the swarm. Update equations:

11 $$\:{v}_{i}\left(t+1\right)={wv}_{i}\left(t\right)+{c}_{1}{r}_{1}\left({P}_{best}-{x}_{i}\left(t\right)\right)+{c}_{2}{r}_{2}\left({G}_{best}-{x}_{i}\left(t\right)\right)$$

12 $$\:{x}_{i}\left(t+1\right)={x}_{i}\left(t\right)+{v}_{i}\left(t+1\right)$$

respectively. In this equation, $\:w$ represents the inertia weight, $\:{c}_{1}$ and $\:{c}_{2}$ are the acceleration coefficients. And $\:{r}_{1}$ and $\:{r}_{2}$ are random values from the (0, 1) interval. The $\:{v}_{i}$and $\:{x}_{i}$ are updated during each iteration. If the maximum number of iterations is achieved or the parameter changes become insignificant, the procedure stops, and the best parameters ($\:s$ and $\:m$) are selected.

### Whale optimization algorithm (WOA)

In this study, WOA—another meta-heuristic optimization method—is also employed as the third method for optimizing the estimation process of WD parameters^25^. Due to its structural characteristics, it is expected to better capture the statistical properties of the continuous $\:H$ data and provide more accurate predictions. The special hunting behavior (hunting called bubble net-feeding) of the humpback whales inspired WOA. Because the position of the optimal design is uncertain, WOA assumes that the target or near-optimal solution is the best choice. Once defined, additional search agents adjust their locations correspondingly, as shown by the equations below^25^.

13 $$\:\overrightarrow{D}=\left|\overrightarrow{C}\:\overrightarrow{{x}^{*}}\left(t\right)-\overrightarrow{x}\left(t\right)\right|$$

14 $$\:\overrightarrow{x}\left(t+1\right)=\overrightarrow{{x}^{*}}\left(t\right)-\overrightarrow{A}\overrightarrow{D}$$

15 $$\:\overrightarrow{A}=2\overrightarrow{a}\overrightarrow{r}-\overrightarrow{a}$$

16 $$\:\overrightarrow{C}=2\:\overrightarrow{r}$$

Where $\:\overrightarrow{D}$ is a vector representing the distance of a search agent to the best solution in the context of WOA. In WOA, each agent moves around the best-known solution ($\:\overrightarrow{{x}^{*}}$) and this movement is updated according to the distance $\:\overrightarrow{D}$. $\:\overrightarrow{A}$ and $\:\overrightarrow{C}$ are coefficient vectors. $\:\overrightarrow{a}$ is linearly decreased from 2 to 0. $\:\overrightarrow{r}$ is a random vector in $\:\left[\text{0,1}\right]$. Whales approach the best solution with logarithmic spiral motion. Here the update equation can be given as follows^25^.

17 $$\:\overrightarrow{x}\left(t+1\right)=\overrightarrow{D{\prime\:}}{e}^{bl}\text{cos}\left(2\pi\:l\right)+\overrightarrow{{x}^{*}}\left(t\right)$$

Where $\:\overrightarrow{D{\prime\:}}=\left|\:\overrightarrow{{x}^{*}}\left(t\right)-\overrightarrow{x}\left(t\right)\right|$. b is a constant value and defines the shape of the logarithmic spiral. l is a random value in $\:\left[-\text{1,1}\right]$.

In addition to swimming in a shrinking circle, humpback whales also follow spiral-shaped paths simultaneously. This next step is to search for prey. In this sense, new solutions are searched for by making random movements using the following equation^25^.

18 $$\:\overrightarrow{x}\left(t+1\right)={\overrightarrow{x}}_{random}\left(t\right)-\overrightarrow{A}\overrightarrow{D}$$

### Long short-term memory (LSTM) networks

**Fig. 2:**
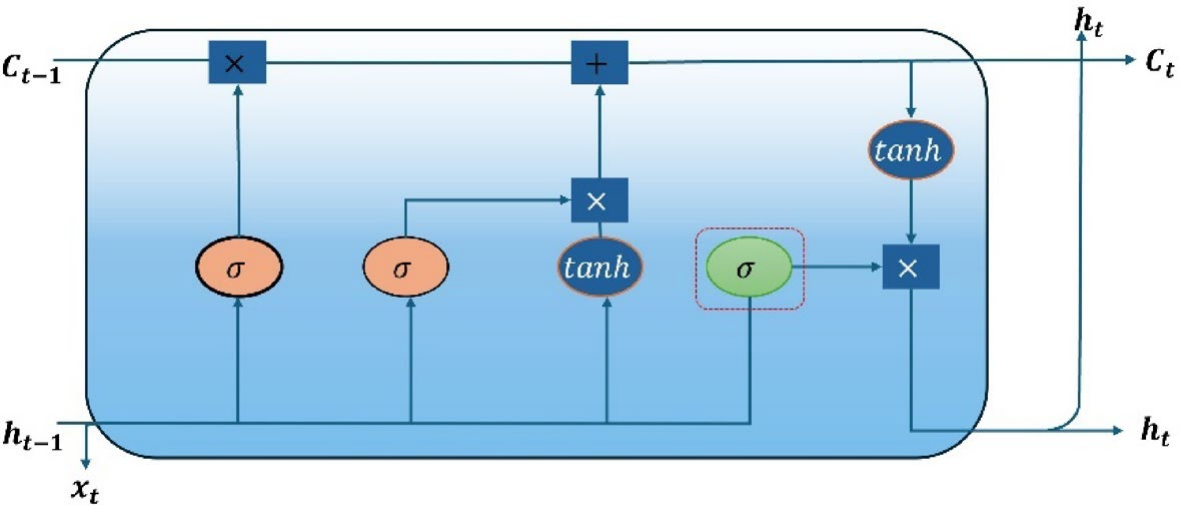
LSTM cell architecture^26^.

**Fig. 3:**
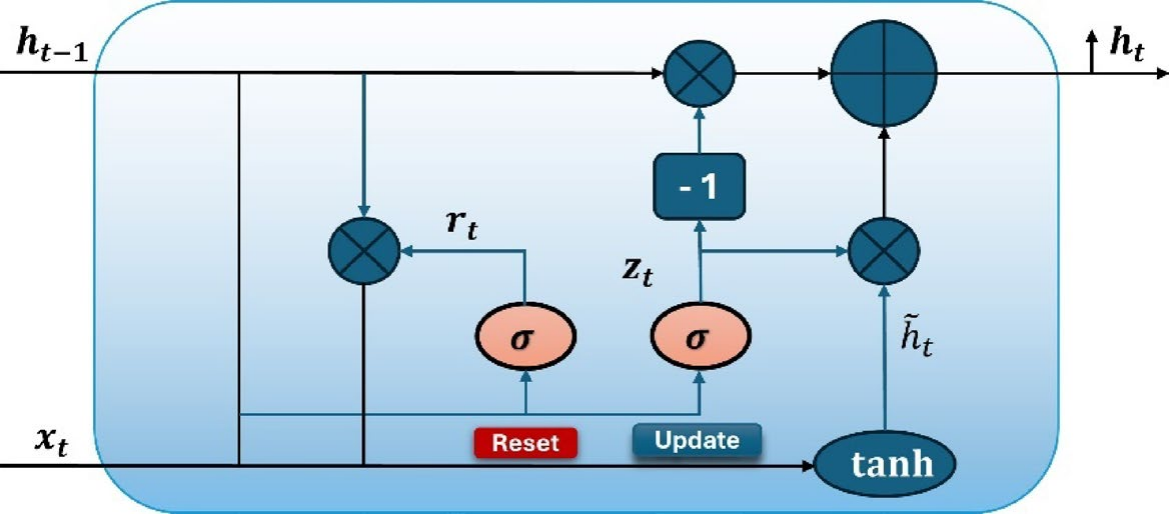
GRU cell architecture^29^.

RNNs are commonly used DL models^26^. However, its fundamental issue is capturing long-term interdependence due to the vanishing gradient problem (VGD)^27^. In^28^, Authors developed LSTM to eliminate VGD. LSTM uses memory cells and gating mechanisms to maintain long-term dependence. Figure 3 depicts the main construction of an LSTM, which contains an input gate, forget gate, cell memory, and output gate. These gates govern and update information within memory blocks. Thus, they allow the model to keep relevant data throughout long sequences. Assume that $\:{x}_{t}$, $\:{h}_{t}$, $\:{c}_{t}$, and $\:{a}_{t}$ represent the input vector, the output vector result at time t, cell statuses at time t, and the update and activation of the current cell status. Furthermore, $\:w$ and $\:b$ denote weight matrices and biases. The calculation of the LSTM is as follows^26^:

19 $$\:{f}_{t}=\sigma\:\left({w}_{f}\left({h}_{t}-1,{x}_{t}\right)+{b}_{f}\right)$$

20 $$\:{i}_{t}=\sigma\:\left({w}_{i}\left({h}_{t}-1,{x}_{t}\right)+{b}_{i}\right)$$

21 $$\:{o}_{t}=\sigma\:\left({w}_{o}\left({h}_{t}-1,{x}_{t}\right)+{b}_{o}\right)$$

22 $$\:{\stackrel{\sim}{c}}_{t}=tanh\left({w}_{a}\left({h}_{t}-1,{x}_{t}\right)+{b}_{a}\right)$$

23 $$\:{c}_{t}={\stackrel{\sim}{c}}_{t}\odot{i}_{t}+{f}_{t}\odot{c}_{t-1}$$

24 $$\:{h}_{t}={o}_{t}\odot\text{t}\text{a}\text{n}\text{h}\left({c}_{t}\right)$$

where $\:{f}_{t}$, $\:{i}_{t}$, and $\:{\stackrel{\sim}{c}}_{t}$, are the output values of forget, input, and output gates. In Eqs. 20–22, the sigmoid function ($\:\sigma\:$) is used as a nonlinear activation function. $\:\odot$ represents the Hadamard product. The output of LSTM is calculated by Eqs. 20–25, ensuring that the previous cell state $\:{c}_{t-1}$ is incorporated correctly in the cell state update equation. In the last step, the weight of each gate is changed using an optimization technique based on the estimated error to ensure effective learning and memory retention in the network. Figure 2 shows the general structure of an LSTM cell.

### Gated recurrent unit (GRU) networks

GRU network represents a simplified and highly efficient variant of recurrent neural networks (RNNs) and outperforms LSTM networks on low-complexity sequences^29^. Unlike LSTM, there is no separate cell state ($\:{c}_{t}$). Instead, the updated hidden state ($\:{h}_{t}$) is stored directly. GRU consists of two gates: a reset gate and an update gate. The update gate renews the network’s existing memory, allowing it to retain substantial data inputs. As seen in Fig. 3, the reset gate erases the network’s active memory and enables it to forget values at each time step.

According to Fig. 3, $\:{x}_{t}$ is the input vector at time $\:t$. $\:{h}_{t}$ is the output vector at time $\:t$. $\:{z}_{t}$ and $\:{r}_{t}$ represent the update gate vector and reset gate vector, respectively. In addition, $\:{\stackrel{\sim}{h}}_{t}$ denotes the candidate activation. The GRU cell, including update gate $\:{z}_{t}$, reset gate $\:{r}_{t}$, hidden state $\:{h}_{t}$, and candidate hidden state $\:{\stackrel{\sim}{h}}_{t}$ with input $\:{x}_{t}$, are calculated as follows^29^:

25 $$\:{z}_{t}=\sigma\:\left({w}_{z}{x}_{t}+{U}_{z}{h}_{t-1}+{b}_{z}\right)$$

26 $$\:{r}_{t}=\sigma\:\left({W}_{r}{x}_{t}+{U}_{r}{h}_{t-1}+{b}_{r}\right)$$

27 $$\:{\stackrel{\sim}{h}}_{t}=tanh\left({w}_{h}{x}_{t}+{U}_{h}\left({r}_{t}\odot{h}_{t-1}\right)+{b}_{h}\right)$$

28 $$\:{h}_{t}=\left(1-{z}_{t}\right)\odot{h}_{t-1}+{z}_{t}\odot{\stackrel{\sim}{h}}_{t}$$

where $\:{W}_{h}$, $\:{W}_{r}$, $\:{W}_{s}$, $\:{U}_{z}$, $\:{U}_{r}$, and $\:{U}_{h}$ are corresponding weights matrices. $\:{b}_{r}$, $\:{b}_{h}$ and $\:{b}_{s}$ are biases.

### Jensen-Shannon divergence (JSD)

In this study, JSD is used to quantitatively analyze the dissimilarity between the probability distributions of observed and predicted values^30^. Before JSD analysis, Kernel Density Estimation (KDE) is used to model the observed and estimated data distributions nonparametrically and to measure the similarity between them more accurately. KDE can be expressed as

29 $$\:\widehat{P}\left(x\right)=\frac{1}{nh}\sum\:_{i=1}^{n}K\left(\frac{x-{x}_{i}}{h}\right)$$

where $\:\widehat{P}\left(x\right)$ is the estimated density function. $\:n$ and $\:h$ are the number of data points and bandwidth (which controls the spread of the kernel), respectively. $\:K$ represents kernel function. Here, Gaussian kernel is preferred as the $\:K$. KDE creates a compatible input for JSD. JSD can be thought of as a symmetric and bounded version of the Kullback-Leibler (KL) divergence. JSD can be given as follows^31^.

30 $$\:JSD\left(P,Q\right)=\frac{1}{2}{D}_{KL}\left(P,\frac{P+Q}{2}\right)+\frac{1}{2}{D}_{KL}\left(Q,\frac{P+Q}{2}\right),\:(0\le\:JSD\le\:\text{l}\text{n}(2\left)\right)\:$$

Where $\:P$ and $\:Q$ are two probability distributions that are compared. $\:{D}_{KL}(P,Q)$ is the Kullback-Leibler divergence.

### Model evaluation

$\:{R}^{2}$ is a statistical measure that shows how much of the variance of the dependent variable is explained by the independent variables. In generally, $\:{R}^{2}$ can take values between 0 and 1. $\:{R}^{2}$ close to 1 indicates that the model has a good fit^32^.

31 $$\:{R}^{2}=1-\frac{{\sum\:}_{i=1}^{n}{({y}_{i}-{\widehat{y}}_{i})}^{2}}{{\sum\:}_{i=1}^{n}{({y}_{i}-\stackrel{-}{y})}^{2}}$$

Where $\:{y}_{i}$ and $\:{\widehat{y}}_{i}$ denote observed and estimated values, respectively. $\:\stackrel{-}{y}$ is average of observed values.

RMSE is the root mean square of the difference between the predicted and observed values and is a measure of error. The smaller the RMSE, the better the model’s predictions^33^.

32 $$\:RMSE=\sqrt{\frac{1}{n}{\sum\:}_{i=1}^{n}{({y}_{i}-{\widehat{y}}_{i})}^{2}}$$

where n is number of observations.

MAPE is the average of the absolute values of the ratio of the prediction errors to the observed values. It is expressed as a percentage. And it is expected to be small in terms of the performance of model^34^.

33 $$\:MAPE=\frac{100\%}{n}\sum\:\left|\frac{{y}_{i}-{\widehat{y}}_{i}}{{y}_{i}}\right|$$

Kling-Gupta Efficiency (KGE) combines correlation, variability ratio, and mean ratio to assess model performance^35^.

$$\:KGE=1-\sqrt{{\left(r-1\right)}^{2}+{\left(\alpha\:-1\right)}^{2}+{\left(\beta\:-1\right)}^{2}}$$

where $\:r$ is the Pearson correlation coefficient, $\:\alpha\:=\frac{{\sigma\:}_{P}}{{\sigma\:}_{O}}$ is the ratio of the standard deviation of predictions to the standard deviation of observations, $\:\beta\:=\frac{\stackrel{-}{P}}{\stackrel{-}{O}}$ is the ratio of the mean of predictions to the mean of observations.

## Results and discussion

In this study, WD is used to analyze the statistical properties of $\:H$ (MJ/m^2^) data and to determine if they follow a statistical pattern. Also, the WD is optimized to better understand the natural structure of data and enrich the inputs of the model. Deterministic MLE and Meta-heuristic WOA and PSO methods are used for optimization. All the computational work is done on a laptop with the following configurations: an Intel Core i7-12700 H@4.70 GHz, 32GB DDR4 RAM (3200 MHz), and an NVIDIA GeForce RTX 3050 (6GB) GPU. Python and DL libraries, such as TensorFlow, Keras, Matplotlib, and Pandas, are used to develop the estimation algorithms as well as the visualization and processing of data. Thus, the developed models are more effective in capturing the basic characteristics of $\:H$ (MJ/m^2^) data.

**Table 1:** Monthly WD parameters estimated by MLE, PSO, and WOA.

Month	MLE		PSO		WOA	
	Shape	Scale	Shape	Scale	Shape	Scale
1	3.7195	8.5278	3.7194	8.5277	0.1191	0.1191
2	4.0614	10.7488	4.0614	10.7488	0.1008	0.1008
3	3.8951	16.2054	3.8952	16.2054	0.4213	0.4213
4	6.2454	20.6737	5.0000	20.0000	0.1226	0.1226
5	6.7103	24.1486	3.5372	20.0000	0.1431	0.1431
6	15.7619	26.0036	3.2814	20.0000	0.1632	0.1632
7	10.4566	23.9086	4.3081	20.0000	0.1175	0.1175
8	22.2362	22.3295	5.0000	20.0000	0.2153	0.2153
9	8.5532	18.2583	5.0000	17.8799	2.9180	11.6602
10	4.2589	14.3637	4.2589	14.3637	0.5392	2.1568
11	5.0197	9.8322	5.0000	9.8295	0.1110	0.1110
12	4.3133	7.0535	4.3133	7.0535	0.7340	1.8724
Annual	7.9360	16.8378	4.2813	15.3840	0.4754	1.4336

Table 1 presents the WD parameters obtained using the MLE, PSO and WOA methods. The highest shape and scale values for all months are calculated by MLE method. The shape parameter of MLE reaches 22.2362 (August) and the scale value reaches 26.0036 (June). The PSO method calculated relatively high shape and scale parameters such as 5.00 for shape (e.g. April, August, September) and 20.00 for scale in more than one month. WeibullCDF values are calculated using WD parameters obtained through MLE, PSO and WOA methods. WeibullCDF and observed $\:H\:$(MJ/m^2^) data are used as input for Weibull-LSTM and Weibull-GRU models. The factors considered during the development of LSTM and GRU models are the network topology, hyperparameters, optimization techniques and data splitting (train-test split ratio of 80:20). Estimation attempts by Weibull - LSTM and Weibull - GRU models are conducted by varying the number of hidden layers from 5 to 100. LSTM and GRU both employed two hidden-layer architectures. These trials allowed for a maximum of 200 epochs. After the data are standardized, each variable is given a uniform weight between 0 and 1. MSE is used as a loss function for all developed models. The learning rate for optimization is set to 0.005, which determines continuous variable improvement parameters. In addition, in the Weibull - LSTM and Weibull - GRU models, ADAM optimizer, an advanced variant of gradient descent, is used as the optimization technique to continuously update network parameters based on the training data. However, Gradient boosting is used during the XGBoost training phase. Since XGBoost is a tree-based model, it uses tree structures instead of layer. It represents the total output as the sum of estimates from all trees. The reg: squarederror (in keras.io) objective allows XGBoost to optimize regression outputs using MSE. Input consists of two features per sample (GRU prediction + Weibull CDF) for XGBoost. XGBoost does not have a temporal dimension (unlike RNNs) and processes individual time points. (5,2) $\:TS$×features explicitly indicate 5 time-steps ($\:TS$-sequence length) and 2 features per $\:TS$($\:H$ + WeibullCDF). Both ultimately produce single-value $\:H$ (MJ/m²) predictions. XGBoost processes point-in-time data (2 features per prediction) and ultimately produces single-value radiation predictions. In the developed models, the batch size is set to 8, and the activation function is set to ReLU. The most successful results are obtained using 50 neurons in the hidden layers (Table 2). In addition, the number of iterations for both deep learning and XGBoost models is determined by the trial-and-error method to ensure the best performance, and it was set to 50. The learning rate used in the deep learning models was 0.005, while the learning rate for the XGBoost model is 0.01. The population size for PSO and WOA is set to 50. PSO’s inertia Weight ($\:{I}_{w}$) is 0.7 and the acceleration coefficients ($\:{c}_{1},{c}_{2}$) were 1.5 and 1.5, respectively, while WOA’s spiral factor ($\:b$) is 1 and the convergence factor ($\:{c}_{f}$) had a decreasing value from 2 to 0. To address the nonlinear relationships that LSTM and GRU missed (especially with Weibull CDF), XGBoost is integrated into Weibull - LSTM and Weibull - GRU models. Thus, temporal and statistical integration is achieved. XGBoost refined the predictions of LSTM and GRU and improved them further.

**Fig. 4:**
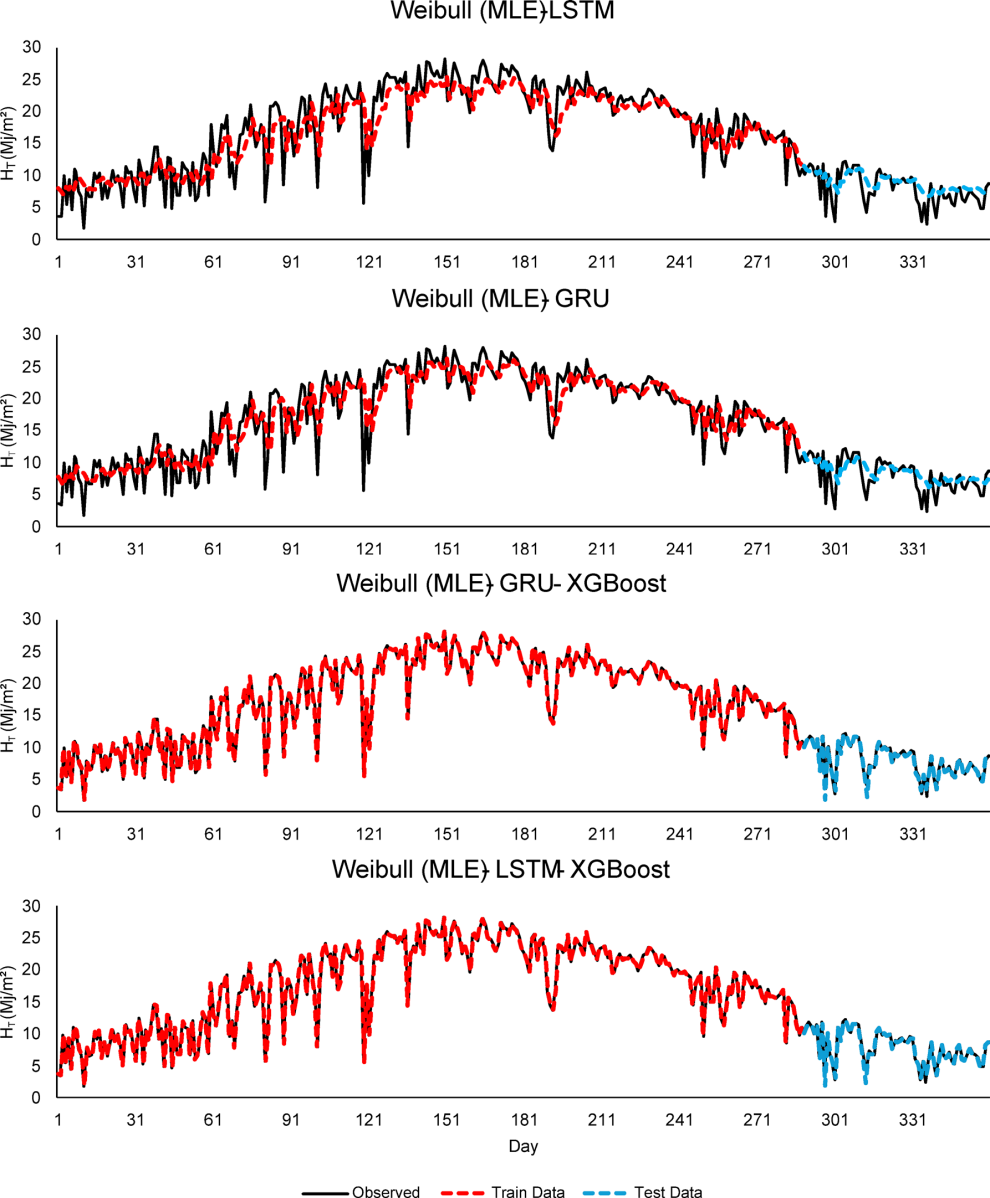
Comparison of LSTM, GRU, and hybrid GRU/LSTM - XGBoost models for $\:H$ (MJ/m^2^) forecasting using Weibull (MLE).

**Fig. 5:**
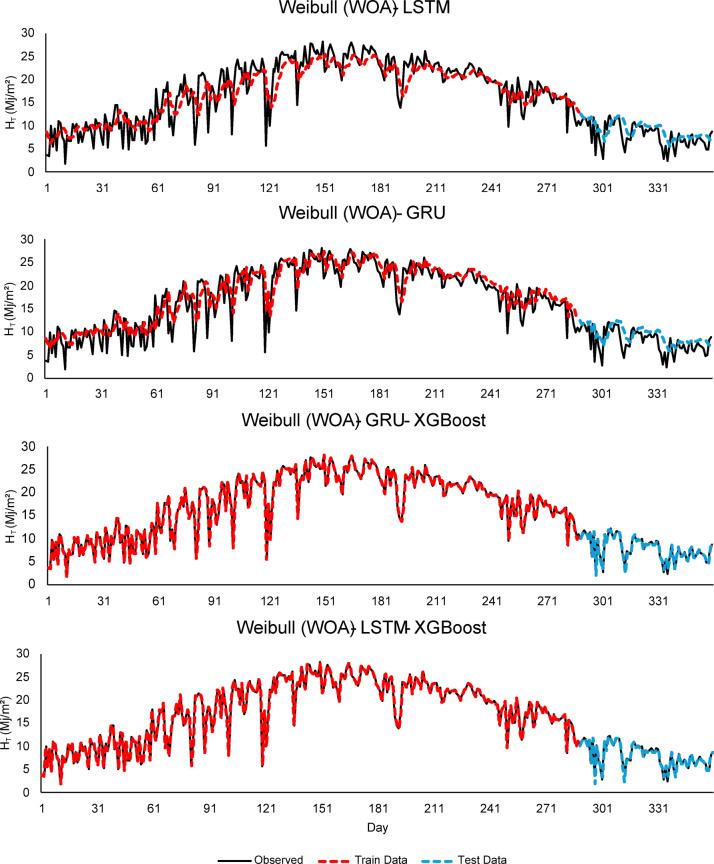
Comparison of LSTM, GRU, and hybrid GRU/LSTM - XGBoost models for $\:H$ (MJ/m^2^) forecasting using Weibull (WOA).

**Fig. 6:**
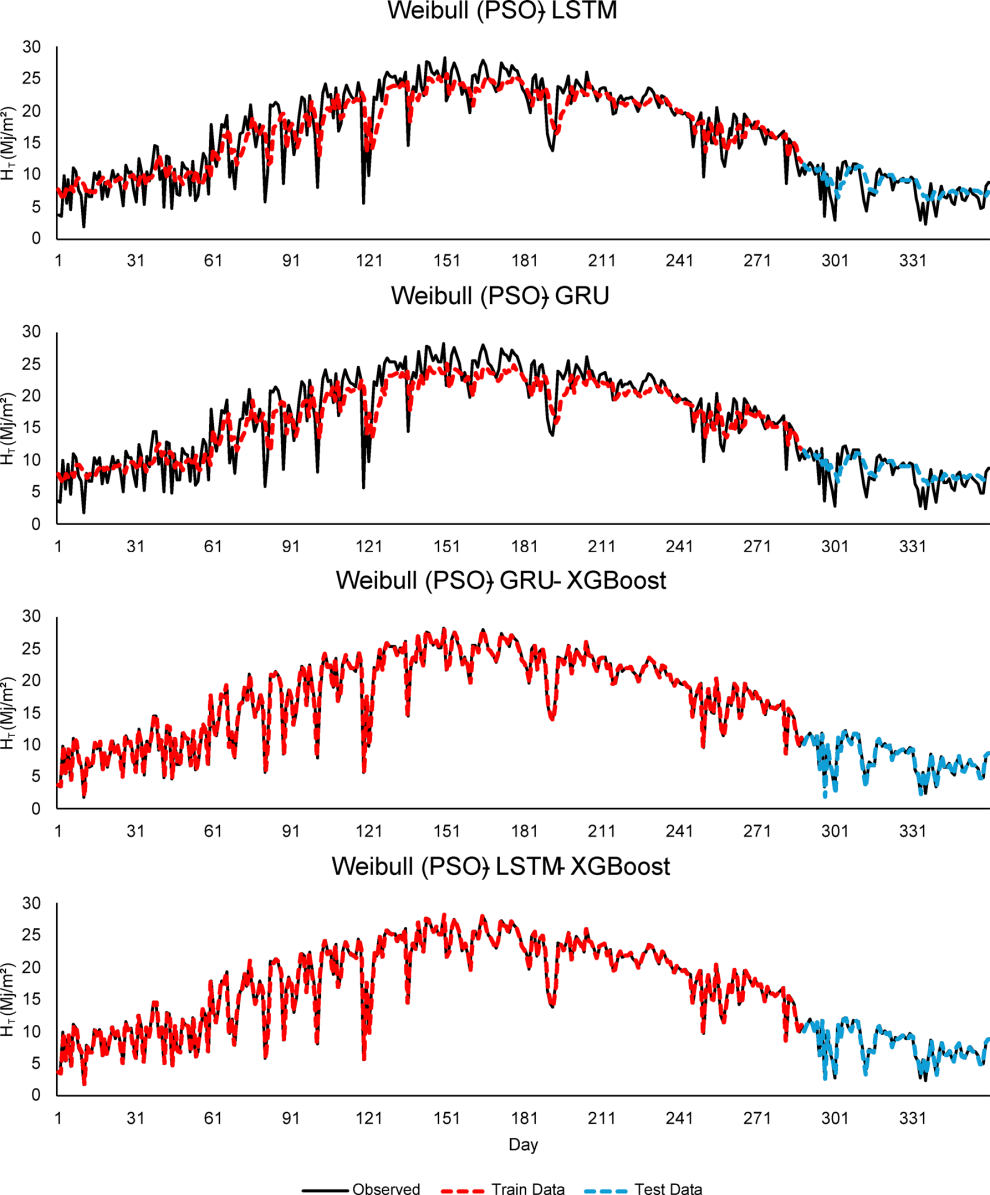
Comparison of LSTM, GRU, and hybrid GRU/LSTM - XGBoost models for $\:H$ (MJ/m^2^) forecasting using Weibull (PSO).

As seen in Figs. 4, 5 and 6, this integration increased the overall performance of the models and provided more accurate results.

**Table 2:** Structural and input feature overview of the developed models.

Parameter	LSTM model	GRU model	XGBoost model
Architecture type	Recurrent	Recurrent	Gradient boosting
Input features	5-step seq (H + WeibullCDF)	5-step seq (H + WeibullCDF)	LSTM/GRU outputs + WeibullCDF
Input shape	(5,2) $\:TS$×features	(5,2) $\:TS$×features	2 features per sample
Layer 1 (units, activation)	50 LSTM, ReLU	50 GRU, ReLU	max_depth = 6
Layer 2 (units, activation)	50 LSTM, ReLU	50 GRU, ReLU	n_estimators = 100
Output (type, activation)	1 Dense, linear	1 Dense, linear	Objective = squarederror

As seen in Fig. 4, the Weibull (MLE) - LSTM model, although there are small deviations in the test data, the overall fluctuation pattern is preserved. The model predicts high radiation values with high accuracy during the summer months. In the Weibull (MLE) - GRU - XGBoost model, thanks to the integration of XGBoost, the predictions follow the observed data more closely. In other words, it can be inferred that the features extracted by the GRU are effectively learned by XGBoost. While the Weibull (MLE) - LSTM - XGBoost model exhibits strong performance, high consistency is observed between the training and test data. As seen in Fig. 5, Weibull (WOA) - GRU - XGBoost and Weibull (WOA) - LSTM - XGBoost models show better prediction accuracy compared to other models. Since the Weibull parameters optimized with WOA represent the distributional structure of the input data well, it can be inferred that the learning capacity of the models has increased. Considering Fig. 5, the Weibull (WOA) - GRU - XGBoost model demonstrates the best generalization in terms of data fit and highest overall model accuracy in the test data. This suggests that WOA enhanced prediction performance both optimizing hyperparameters and also by improving the structural quality of the input data. PSO effectively reflected the structural characteristics of the data and increased predictive power in the models (seen in Fig. 6). The best performances are observed in the Weibull (PSO) - GRU - XGBoost and Weibull (PSO) - LSTM - XGBoost hybrid models. Both models show high accuracy and low deviation in training and test data.

Considering the classical point prediction metrics, the Weibull (PSO) - LSTM - XGBoost model demonstrates superior point forecasting performance. As presented in Table 3, this model achieves the highest R² (0.9882), the lowest RMSE (0.2595), and the lowest MAPE (0.0263), indicating its exceptional accuracy in predicting individual data points. However, for the primary objective of this study—accurately capturing the probability distribution of the data—the distributional similarity metric (JSD) is deemed more critical, as discussed in the following sections.

While classical point prediction metrics such as $\:{R}^{2}$ and RMSE provide a valuable assessment of point forecast accuracy, the primary evaluation criterion in this study is the Jensen-Shannon Divergence (JSD). This choice is motivated by the core objective of presented modeling approach: not only to achieve accurate point forecasts but to accurately learn and replicate the underlying probability distribution of the solar radiation data. JSD quantitatively measures the similarity between the probability distributions of the predicted and actual data, providing a holistic assessment of distributional fit. This is paramount for applications like solar energy system design and risk assessment, where understanding the full distribution—including the likelihood of extreme values (e.g., periods of very high or very low radiation)—is often more critical than the accuracy of a single point prediction. A model with a low RMSE might still fail to capture the true variability and shape of the data, leading to suboptimal system design decisions. This limitation of point prediction metrics is well-documented in the literature, where metrics like RMSE and R² are traditionally used for model selection. However, if a model fails to mimic the true distribution of the data, it can misrepresent critical features such as fluctuations and extreme values, which R² is not designed to capture. For this reason, the distribution-based JSD metric is adopted in this study as the primary criterion for evaluating how well a model replicates the observed distribution.

**Table 3:** Performance comparison of all models based on point prediction accuracy and distributional similarity metrics.

Model	Type	$\:{R}^{2}$	RMSE	MAPE	KGE	JSD
Weibull (MLE) - LSTM	DOD	0.0135	2.7989	0.3722	0.0167	0.3563
Weibull (MLE) - GRU	DOD	-0.0657	2.9091	0.3791	-0.0543	0.3070
Weibull (MLE) - GRU - XGBoost	DOD-Boost	0.9802	0.3966	0.0383	0.9541	0.0086
Weibull (MLE) - LSTM - XGBoost	DOD-Boost	0.9851	0.3438	0.0399	0.9228	0.0103
Weibull (WOA) - LSTM	DOD	-0.2433	2.6686	0.3124	0.1690	0.2385
Weibull (WOA) - GRU	DOD	-0.3094	2.7386	0.3248	0.1698	0.2594
Weibull (WOA) - GRU - XGBoost	DOD-Boost	0.9726	0.3958	0.0359	0.9304	0.0093
Weibull (WOA) - LSTM - XGBoost	**DOD-Boost**	0.9665	0.4382	0.0382	0.9186	**0.0084**
Weibull (PSO) - LSTM	DOD	-0.0093	2.4043	0.2710	0.1595	0.2497
Weibull (PSO) - GRU	DOD	0.0151	2.3751	0.2710	0.1155	0.2892
Weibull (PSO) - GRU - XGBoost	DOD-Boost	0.9805	0.3344	0.0322	0.9191	0.0100
Weibull (PSO) - LSTM - XGBoost	**DOD-Boost**	**0.9882**	**0.2595**	**0.0263**	**0.9626**	0.0121

**Fig. 7:**
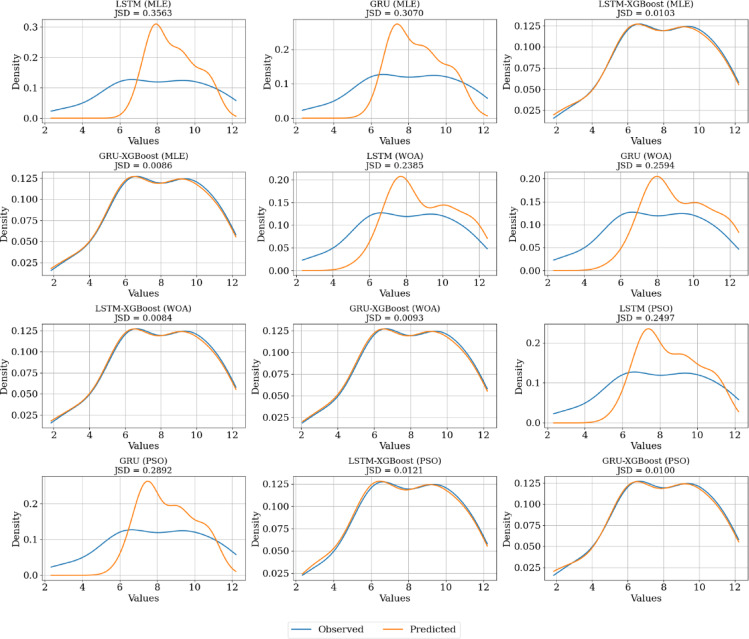
Comparing the performance of models based on KDE distributions and JSD values.

Consistent with the primary objective of this study, the Weibull (WOA) - LSTM - XGBoost model achieves the best distributional fit, as evidenced by its lowest JSD value of 0.0084 (Table 3). This key result indicates that this model most accurately replicates the underlying probability distribution of the solar radiation data, which is critical for probabilistic applications like solar energy system planning and risk assessment. It is important to note that a trade-off exists between point prediction accuracy and distributional similarity. This is demonstrated by the Weibull (PSO) - LSTM - XGBoost model, which excels in point forecasts (highest R² = 0.9882, lowest RMSE = 0.2595) but has a slightly higher JSD (0.0121). Conversely, the superior distributional model (WOA) still maintains highly competitive point prediction accuracy (RMSE = 0.4382, MAPE = 0.0382). This trade-off is not a contradiction but rather a key finding of this study, revealing that different optimization algorithms (PSO vs. WOA) within the hybrid framework prioritize different aspects of model performance. The Weibull (MLE)-GRU-XGBoost model (JSD = 0.0086) further confirms this pattern, achieving excellent distributional similarity despite a marginally lower R² value. The relatively small JSD differences between the top models (Fig. 7) highlight the overall effectiveness of the DOD-Boost approach in achieving distributional fidelity. Therefore, model selection should be guided by the specific application: the Weibull (PSO) - LSTM - XGBoost model is recommended for point forecasts, while the Weibull (WOA) - LSTM - XGBoost model is superior for applications requiring a complete probabilistic understanding of the data.

In contrast, the standalone deep models such as Weibull (WOA) - LSTM and Weibull (WOA) - GRU exhibit poor performance (R² < 0, RMSE > 2.6), but their accuracy improves significantly when XGBoost is incorporated into the framework (i.e., DOD-Boost). As clearly demonstrated in Table 3, all DOD-Boost models consistently outperform their corresponding DOD versions regardless of the optimization technique applied. Among the optimization methods, MLE- and PSO-based models generally yield more consistent and accurate predictions than those based on WOA, especially when evaluated using KGE and MAPE. This suggests that the choice of parameter optimization technique notably influences the prediction capability of both deep and hybrid models.

**Fig. 8:**
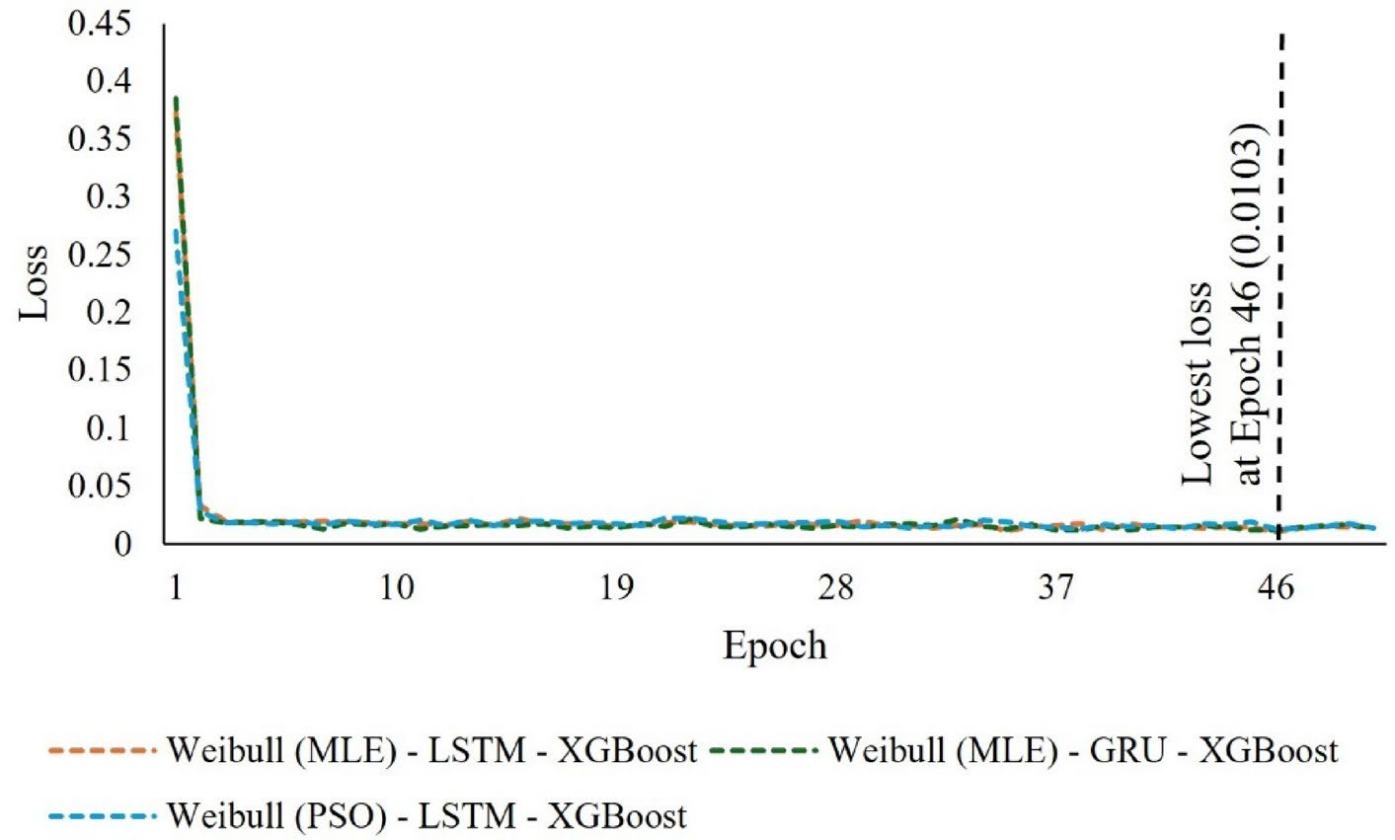
The loss curves of the proposed model during the training phase.

In comparison to the other models (Fig. 8), the Weibull (MLE) - LSTM - XGBoost model has the lowest training loss value of 0.0103 in training phase. This indicates a high degree of predictive potential. While the learning curve of the Weibull (MLE) - GRU - XGBoost model remains relatively steady through 50 epochs, it has been shown to be more robust to $\:H$ (MJ/m^2^) data. The Weibull (MLE) - LSTM - XGBoost is learning faster than the other two models. This is the key indicator that the model is learning effectively. The performance of a model often aims to decrease the loss value, which is evident in Fig. 8.

**Fig. 9:**
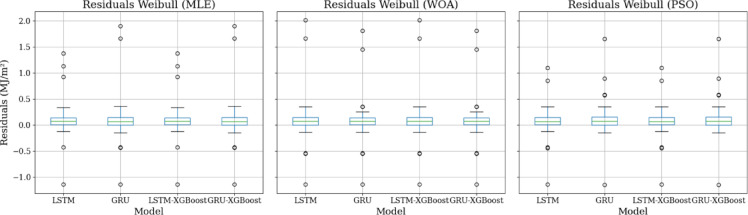
Residual plot analysis for proposed models.

Figure 9 depicts the residuals (predicted - observed) of three models: Weibull (MLE) - GRU - XGBoost, Weibull (MLE) - LSTM - XGBoost, and Weibull (PSO) - LSTM - XGBoost, plotted against observed $\:{H}_{T}$ (MJ/m²) levels. While all models show a reasonably balanced distribution around zero, irregularities become more obvious at lower and higher radiation levels. Weibull (MLE) - LSTM - XGBoost outperforms the other models, with residuals concentrated in a more restrictive band and more consistent estimates throughout the observed values.

As seen in Table 4, the results suggest that the results of the present study using the DOD-Boost framework are successful when compared to previously published studies. While the studies conducted by Azizi et al. and Wentz et al. obtained high $\:{R}^{2}$ values around 0.95, the DOD-Boost model obtained a higher $\:{R}^{2}$ value of 0.9896. Additionally, the proposed model achieved an RMSE of 7.0808 MJ/m^2^ against the RMSE values reported in other studies. These findings indicate that proposed models in the DOD-Boost framework increase their ability to estimate solar radiation with the integration of statistical preprocessing and hybrid DL.

**Table 4:** Comparative performance analysis between the present study and published studies.

Author	Models	RMSE (W/m^2^)	$\:{R}^{2}$
Sansine et al.^35^	LSTM	184.7400	0.7400
	Gradient Boosting	174.5400	0.7700
	XGboost	176.4700	0.7600
	PSO-Gradient Boosting	167.2400	0.7900
	PSO-LSTM	154.8400	0.8200
	PSO-XGboost	153.6900	0.8200
Azizi et al.^36^	LSTM_I	13.3200	0.9548
	LSTM_II	13.6400	0.9525
	GRU_I	14.2200	0.9484
	GRU_II	13.0800	0.9563
Wentz et al.^37^	LSTM	0.097 (nRMSE)	0.8560
Blazakis et al.^38^	LSTM	0.05–0.72 (nRMSE)	0.71–0.97
Present study	DOD-Boost (Weibull (PSO) - LSTM - XGBoost)	7.0808 (MJ/m^2^)	0.9882

**Fig. 10:**
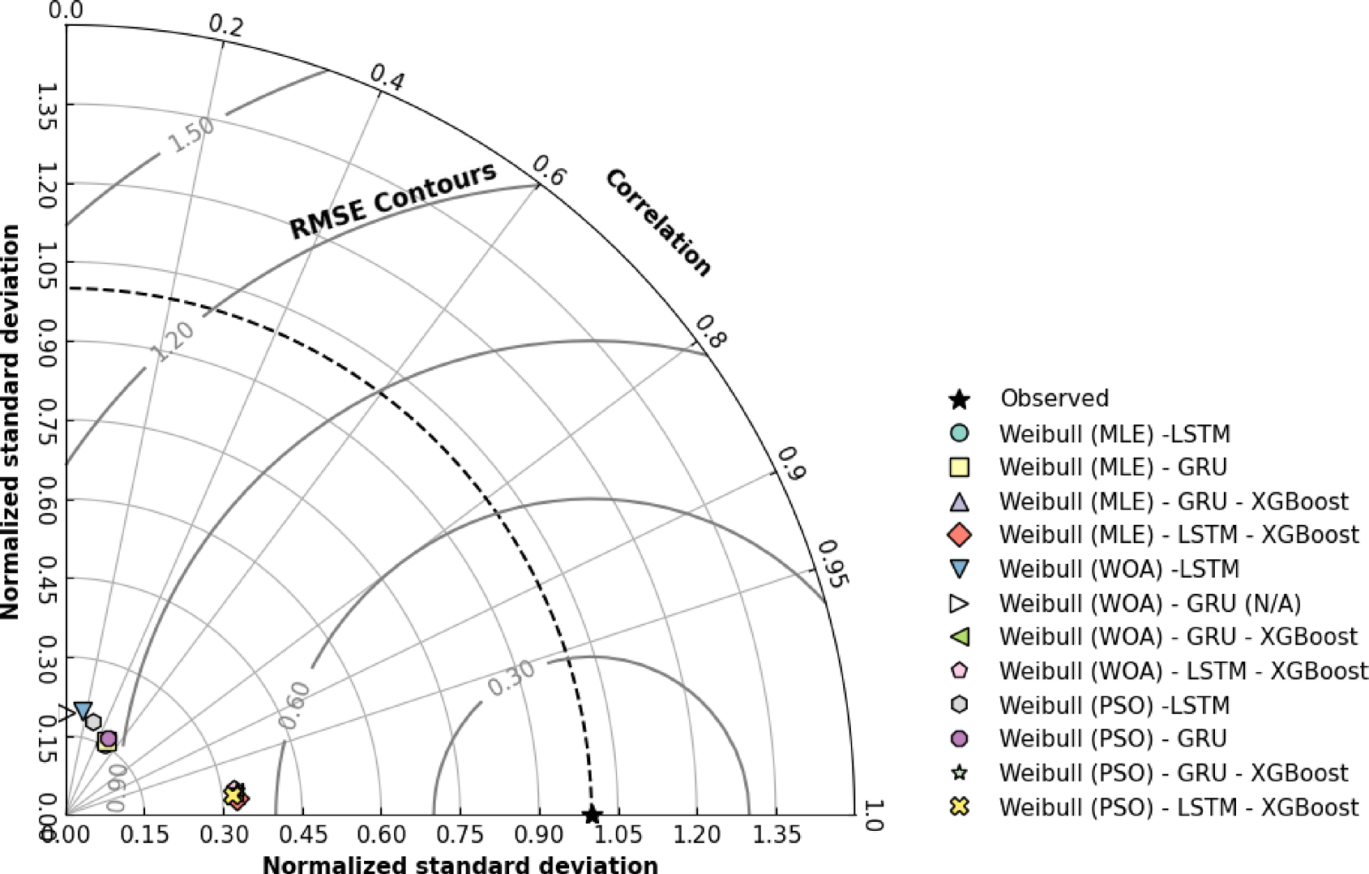
Taylor diagram analysis.

In this study, the proposed models are evaluated by the Taylor diagram and statistical metrics. As seen in Fig. 10, in the Taylor diagram, correlation coefficients increase to the 0.99 level (*p* < 0.001) and normalized RMSE values decrease from 60 to 70 range to 7–10 range. Among all, MLE-LSTM-XGBoost model is the most successful model, with a correlation value of 0.995 and a 98% variance explanation ratio. The findings indicate that DOD-Boost approach increases model performance in all optimization methods (MLE, WOA, PSO) at a statistically significant level (*p* < 0.001). Performance differences between MLE, WOA, and PSO are significant when XGBoost (*p* = 0.023) is excluded from models. The effect of these algorithms decreased significantly with the inclusion of XGBoost in the model (*p* = 0.451). However, it quantitatively demonstrates how DOD and DOD-Boost approaches increase statistical reliability in parametric estimation problems.

**Fig. 11:**
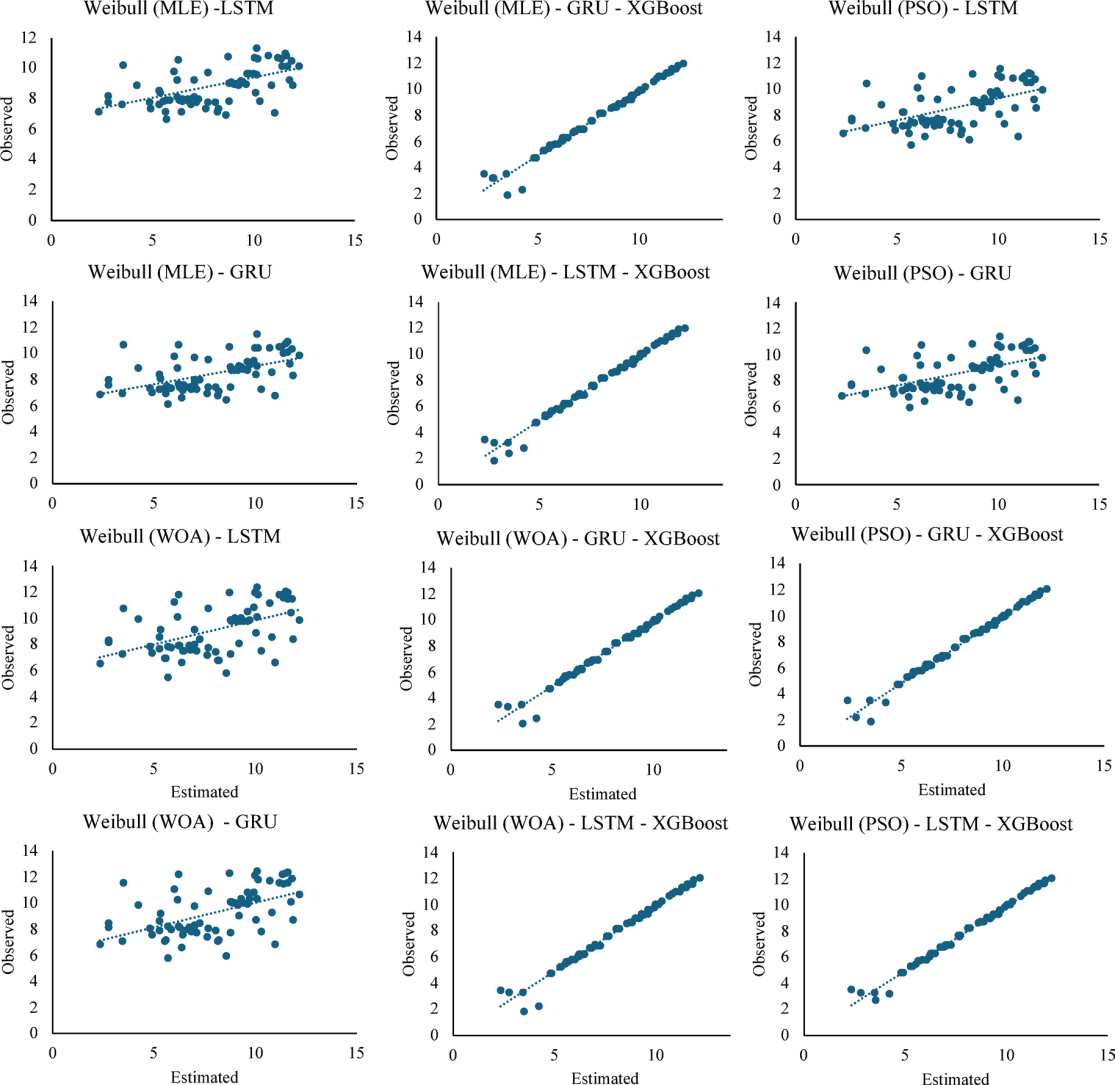
Comparison of $\:{H}_{T}$ values using Weibull (MLE-WOA-PSO)-based hybrid models.

Figure 11, compare observed and estimated $\:{H}_{T}$ values using Weibull (MLE-WOA-PSO) with LSTM, GRU, and XGBoost-based hybrid models on test data.

## Conclusion

This study proposes a novel DOD-Boost framework for solar power potential based on daily $\:{H}_{T}$ (MJ/m^2^) data obtained from TSMS. The DOD-Boost framework integrates three methodological components: The first is robust distributional feature extraction through WeibullCDF using parameters optimized via MLE, WOA, and PSO. The second is deep temporal modeling using LSTM and GRU networks to capture $\:H$ (MJ/m^2^) patterns. Ensemble performance improvement by XGBoost for residual correction and feature importance-weighted prediction is the third.

Developing the models follows two distinct strategies: First strategy: Developing Weibull (MLE/WOA/PSO) - LSTM/GRU models as the DOD framework. WeibullCDF and observed $\:{H}_{T}$ (MJ/m^2^) values serve as inputs to the LSTM/GRU models for estimating $\:{H}_{T}$ (MJ/m^2^). Second strategy: The XGBoost model is integrated with Weibull (MLE/WOA/PSO) - LSTM/GRU models. Thus, the DOD-Boost framework is constructed. In this strategy, the outputs of the LSTM/GRU models and WeibullCDF values are used as inputs for XGBoost to estimate $\:{H}_{T}$ (MJ/m^2^). Under the first strategy, developed models based on the DOD framework show limited accuracy.

The standalone deep learning models (DOD), such as Weibull (MLE) - LSTM (RMSE: 2.7989 MJ/m², MAPE: 0.3722) and Weibull (PSO) - GRU (RMSE: 2.3751, R²: 0.0151), demonstrate significantly weaker performance compared to the hybrid DOD-Boost models. This result clearly highlights the limitation of relying solely on deep networks for this task.

The RMSE of the Weibull (PSO) - GRU model is 2.3751, MAPE is 0.2710, and $\:{R}^{2}$ is 0.0151. In the second strategy, all models outperform their earlier counterparts.

By comparing the test results, LSTM-based models within the DOD-Boost framework generally provide more accurate point forecasts for $\:{H}_{T}$ (MJ/m^2^) than GRU-based models, as reflected in improved R², RMSE, and MAPE values. Specifically, the Weibull (PSO) - LSTM - XGBoost model achieves the best point prediction performance.

However, according to the primary evaluation criterion of this study, the Jensen-Shannon Divergence (JSD), the Weibull (WOA) - LSTM - XGBoost model achieves the best distributional fit with the lowest JSD value (0.0084). This is a key and novel finding of presented work, as it demonstrates the model’s superior ability to capture the underlying probabilistic nature of solar radiation data, moving beyond traditional point prediction metrics. The Weibull (MLE) - GRU - XGBoost model also exhibits excellent performance, finishing a very close second (JSD = 0.0086). The minimal difference between their JSD values reinforces that the proposed DOD-Boost framework is highly effective in preserving the original data’s distributional fidelity, with the WOA-optimized LSTM variant being the top performer.

The successful integration of these divergence-based metrics provides a more comprehensive assessment of model performance, moving beyond simple point-wise error measures to capture the full distributional behavior of the data. As a result, the proposed DOD-Boost framework, which successfully integrates hybrid modeling approaches, optimization techniques, data preprocessing strategies, and temporal modeling, overcomes the limitations of a single model and provides both highly accurate point predictions and probabilistic meaning to the data.

## Data Availability

The data that support the findings of this study are available from the corresponding author, Dr. İlker Mert (ilkermert@osmaniye.edu.tr), upon reasonable request.

## Abbreviations

ANN: Artificial neural network
CDF: Cumulative distribution function
DL: Deep learning
DOD-Boost: Distribution-optimized deep boosting
GRU: Gated recurrent unit
JSD: Jensen-Shannon divergence
KDE: Kernel density estimation
KGE: Kling-Gupta efficiency
LSTM: Long short-term memory
MAPE: Mean absolute percentage error (%)
ML: Machine learning
MLE: Maximum likelihood estimation
NLL: Negative log-likelihood
PSO: Particle swarm optimization
R^2^: Coefficient of determination
RMSE: Root mean square error (MJ/m^2^)
SMS: State meteorological service
SVR: Support vector regression
TSD: Time series data
WD: Weibull distribution
WOA: Whale optimization algorithm
XGBoost: Extreme gradient boosting

## List of symbols

$H$: Total solar radiation on a horizontal surface (MJ/m^2^)
${H}_{T}$: Total solar radiation on a tilted surface (MJ/m^2^)
${H}_{d}$: Diffuse component of total solar radiation (MJ/m^2^)
${H}_{\rho\:}$: Reflected component of total solar radiation (MJ/m^2^)
$s$: Scale parameter of the Weibull distribution
$m$: Shape parameter of the Weibull distribution
$\beta$: Slope angle of the surface (Degrees)
$g\left(x\right)$: Weibull cumulative distribution function
$G\left(x\right)$: Weibull probability density functions (Weibull PDF)
${H}_{Weibull}$: Expected daily $H$ (MJ/m^2^) based on $G$ (MJ/m^2^)
$$p$$: Probability value
$r$: Pearson correlation coefficient
${\stackrel{-}{R}}_{b}$: Ratio of direct radiation on a tilted vs. horizontal surface

## Greek symbols

$\varphi$: Latitude angle of the surface (Degrees)
$\delta$: Declination angle of the surface (Degrees)
${\omega}_{s}$: Sunset hour angle on a horizontal surface (Degrees)
${{\omega}^{\prime}}_{s}$: Sunset hour angle on a tilted surface (Degrees)
$\alpha$: Ratio of the standard deviation of predictions to observations
